# Renin-Angiotensin-Aldosterone System in Women Using Combined Oral Contraceptive: A Systematic Review

**DOI:** 10.1055/s-0042-1745790

**Published:** 2022-06-20

**Authors:** Priscilla Araújo dos Santos, Alice Miranda de Oliveira, Caroline Queiroz Alves, Clóvis Figueiredo Souza Filho, Ana Marice Teixeira Ladeia, Jefferson Petto

**Affiliations:** 1 Escola Bahiana de Medicina e Saúde Pública, Salvador, BA, Brazil; 2 Actus Cordios Reabilitação Cardiovascular, Respiratória e Metabólica, Salvador, BA, Brazil; 3 Centro Universitário UniFTC, Salvador, BA, Brazil

**Keywords:** contraceptives, blood pressure, renin-angiotensin system, hypertension, women's health, anticoncepcionais, pressão arterial, sistema renina-angiotensina, hipertensão, saúde da mulher

## Abstract

**Objective** To describe the effects of combined oral contraceptives (COC) on the renin-angiotensin-aldosterone system (RAAS).

**Data sources** This is a systematic review according to the criteria of Preferred Reporting Items for Systematic Reviews and Meta-Analyses (PRISMA), registered in PROSPERO under the ID: CRD42020200019. Searches were performed between August 2020 and December 2021, in the following databases: Medline via Pubmed, Cochrane Central Library, Scientific Electronic Library Online, and Latin American and Caribbean Literature in Health Sciences via Virtual Health Library. The effects of the combined oral contraceptive on plasma renin activity values, plasma renin values, angiotensinogen values— also known as plasma renin substrate— angiotensin, and/or aldosterone values.

**Study selection** A total of 877 studies were selected and, of these, 10 articles met the eligibility criteria and were included in this review.

**Data collection** Data were combined through qualitative synthesis and included in a spreadsheet previously prepared by the authors.

**Data synthesis** The collected samples ranged from 18 to 137 participants, totaling 501 women aged between 18 and 49 years throughout all studies. The studies showed increased activity of plasma renin, plasma renin substrate, angiotensin II, and aldosterone in this population.

**Conclusion** The findings of this study suggest that the COC promotes greater activation of the RAAS. Supporting the idea that its use is related to an increased risk of cardiovascular events, including systemic arterial hypertension.

## Resumo

**Objetivo** Descrever os efeitos do contraceptivo oral combinado (COC) no sistema renina-angiotensina-aldosterona (SRAA).

**Fontes dos dados** Trata-se de uma revisão sistemática de acordo com os critérios do Preferred Reporting Items for Systematic Reviews and Meta-Analyses (PRISMA), registrada no PROSPERO sob ID: CRD42020200019. As buscas foram realizadas entre agosto de 2020 e dezembro de 2021 nas bases de dados: Medline via Pubmed, Biblioteca Cochrane Central, Scientific Electronic Library Online, e Literatura Latino-americana e do Caribe em Ciências da Saúde via Biblioteca Virtual em Saúde. Consultado os artigos sobre os efeitos do contraceptivo oral combinado nos valores da atividade da renina plasmática, valores plasmáticos da renina, valores do angiotensinogênio – também conhecido como substrato da renina plasmática –, valores da angiotensina e/ou aldosterona.

**Seleção dos estudos** Foram selecionados 877 estudos e, destes, 10 artigos preencheram os critérios de elegibilidade e foram incluídos nesta revisão.

**Coleta de dados** Os dados foram combinados por meio de síntese qualitativa e inclusos em uma planilha elaborada previamente pelos autores.

**Síntese dos dados** As amostras coletadas variavam entre 18 e 137 participantes, totalizando 501 mulheres com idade entre 18 e 49 anos em todos os estudos. Os estudos apresentaram aumento da atividade da renina plasmática, do substrato da renina plasmática, da angiotensina II e da aldosterona nessa população.

**Conclusão** Os achados deste estudo sugerem que o COC promove maior ativação do SRAA. Apoiando a ideia de que o seu uso esteja relacionado ao aumento do risco de eventos cardiovasculares, incluindo a hipertensão arterial sistêmica.

## Introduction

The adverse effects of the use of the combined oral contraceptive (COC) have been the subject of much research, including the relationship of the COC in the development of systemic arterial hypertension (SAH). 1 2 3 4 One of the main mechanisms that explain this relationship is the action of COC on the renin-angiotensin-aldosterone system (RAAS). 5

A review carried out by Oelkers 5 in 1996 proposed to report the effects of estrogens and progestogens that compose the COC on the RAAS and on the blood pressure (BP). This study pointed out that the estrogenic and progestogenic components are responsible for the activation of the RAAS and may explain the increase in BP in this population. Corroborating this study, a randomized clinical trial showed that women using COC showed a significant increase in the values of plasma renin activity (PRA) when compared to the control group. 6

More recently our research group has verified through an observational study that women using COC had a median 2-fold higher plasma renin than women not using COC. 7 In this sense, based on the fact that the RAAS is an important physiological regulator of BP and that the use of COC may be indicated as a generator and sustainer of high blood pressure levels, it is necessary to understand how the RAAS may promote the elevation of BP in users of COC and what the studies show about this relationship. Therefore, this study aims to describe the effects of COC on the RAAS.

## Methods

This is a systematic review according to the criteria of the Preferred Reporting Items for Systematic Reviews and Meta-Analyses (PRISMA). 8 The searches were conducted between August 2020 and December 2021 in the following databases: Medline via PubMed, Cochrane Central Library, Scientific Electronic Library Online (Scielo), and Latin American and Caribbean Literature on Health Sciences (Lilacs) via Virtual Health Library (VHL). References of the selected papers were also checked to find other studies related to the topic. This review was registered in PROSPERO under id: CRD42020200019.

We considered eligible original studies with control or comparison groups, which evaluated young, healthy women of reproductive age (> 18 years), who were users of COC. The outcomes observed in the studies had to involve the effects of COC on the values of plasma renin activity (PRA), plasma renin values, angiotensinogen values also called plasma renin substrate (PRS), angiotensin and/or aldosterone values, as well as the mechanisms related to their alterations.

Studies with menopausal women, women with cardiovascular diseases or metabolic disorders were not eligible. Studies with obese women, smokers, drinkers, or those undergoing drug treatment were also excluded.

For the search, the Medical Subject Headings (MeSH) terms *Contraceptives, Oral* AND *Renin-Angiotensin System* were crossed with their respective synonyms. In the Portuguese language databases, the same searches were repeated using the Health Science Descriptors (DeCS). No restrictions on publication period and no language restrictions.

Search and screening of the articles was performed independently by two reviewers, initially by the titles and abstracts. Subsequently, all articles that met the selection criteria were chosen, and the full text was read. Duplicates were identified and removed using the Rayyan QCRI (Rayyan Systems Inc. Cambridge, MA, EUA) web/mobile application. 9 In case of disagreement about the selection of studies, the decision was discussed among the researchers.

The data from the selected studies were combined by means of qualitative synthesis. Therefore, after confirming the selected articles, the data were assigned to a spreadsheet previously prepared by the authors. Disagreements about the extracted data were discussed among the researchers.

The risk of bias assessment of clinical trials was performed by the Cochrane Collaboration Tool (Cochrane Collaboration, London, UK). 10 This tool critically assesses the risk of bias in studies through 7 domains, namely: random sequence generation, allocation concealment, blinding of participants and professionals, blinding of outcome evaluators, incomplete outcomes, selective outcome reporting, and other sources of bias.

The quality of evidence from observational studies was assessed using the Downs and Black 11 scale whose assessment includes communication (reporting), external validity, internal validity (bias, confounding variables), and statistical power. In each evaluation (except for question 5, in which the maximum value is 2) a score of 0 was assigned for conditions not presented in the study and 1 for identified criteria. Two researchers participated in this step and any differing results would be evaluated by a third researcher, but there was no need.

## Results

**Fig. 1:**
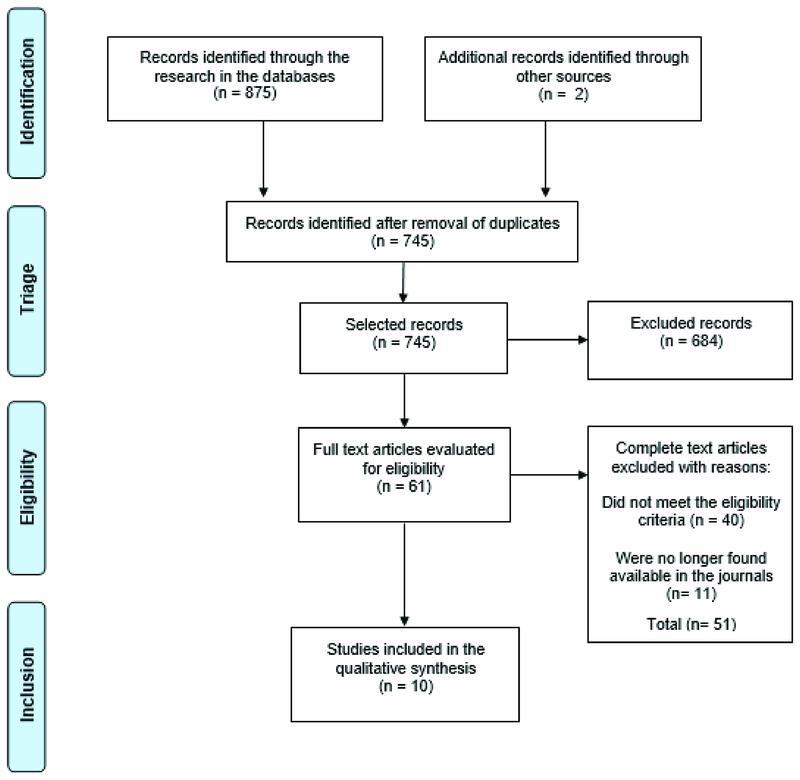
Study selection flowchart.

The selection in the databases resulted in 875 articles. Two articles were identified through the references, resulting in 877 articles; of these, ten studies met the eligibility criteria and were included in this review. Figure 1 shows the flowchart of study selection.

The samples of the selected studies comprised 18 to 137 women, with a total of 500 patients throughout all studies, aged 18 to 49 years. Of the ten studies included, four were clinical trials and six were observational studies. The characterization of the studies is presented in Chart 1 .

**Chart 1:** – Qualitative synthesis of the selected studies

Author, year	Objective	Study design	Sample	Intervention or exposure protocol	Method (RAAS)	Results
Straznicky et al. (1998) 6	To prospectively investigate the interaction effects of COC use and short-term dietary fat intake on 24 hour ambulatory BP, cardiovascular reactivity, and glucose tolerance.	Randomized and Crossover Clinical Trial	Total: 31 women Age: 18–45 years old	Experimental diets were designed to produce maximal changes in plasma low-density lipoprotein cholesterol concentration. Investigations were carried out on the last 2 days of each diet, which were scheduled to coincide with days 15–21 of the pill intake cycle (for the 16 COC users) or days 21–27 of the menstrual cycle (16 volunteers in the control group).	PRA: measured by incubating plasma for 3h in the presence of converting enzyme inhibitors and angiotensinases (EDTA and phenylmethylsulfonyl fluoride).	PRA was significantly higher in COC users than in non-users with both low-fat (2.0 ± 0.8 vs. 1.3 ± 0.7ng/ml per hour, *p* = 0.02) and rich diet in fat (1.9 ± 0.6 versus 1.3 ± 0.8ng/ml per hour, *p* = 0.03), and was not influenced by dietary change.
Briggs and Briggs (1982) 12	To compare the metabolic impact of different oral contraceptive formulations on new oral contraceptive acceptors in healthy young women.	Prospective Randomized Clinical Trials	Total: 137 women Age: < 30-years-old	Study 1: The volunteers were randomized to 6 cycles of treatments in one of 4 groups: Group 1: 0.15mg LNG + 30mcg EE. Group 2: 0.50mg NET + 35mcg EE. Group 3: 1.00mg NET + 35mcg EE. Group 4: 2.00 mg EDA + 30 mcg EE. Two blood samples were taken on consecutive days during the late pretreatment cycle (days 25–28) and during each treatment cycle on any of the last two days of tablet ingestion. Study 2: Participants volunteers were randomized to receive the following formulations for 12 treatment cycles: Single-phase – (x 21) 0.150mg LNG and 30mcg EE. Single-phase – (x 21) 0.250mg LNG and 50mcg EE. Biphasic – (x 10) 0.050mg LNG and 50mcg EE; (x 11) 0.125mg LNG and 50mcg EE. Three-phase – (x 6) 0.050mg LNG and 30mcg EE; (x 5) 0.075mg LNG and 40mcg EE; (x 11) 0.125mg LNG and 30mcg EE. Measurements of PRA, PRS, and renin concentration were performed pre-treatment and every 6 cycles.		With the administration of the two products with the highest dose of estrogen ( 0.250mg LNG + 50 mcg EE, and the biphasic), PRA and PRS concentration approximately doubled ( *p* < 0.001). In contrast, renin concentration was reduced by about 30–40% from pretreatment values ( *p* < 0.001) for the same formulations. Changes with the two lower dose products were minor but still statistically significant ( *p* < 0.01). The increase in PRA was 20–30%, the PRS concentration 12–20%, while the drop in renin concentration was about 15–20%.
Cain et al. (1971) 13	Evaluate the levels of angiotensin II, PRA, PRS, and renin concentration during treatment with estrogen and progestin oral contraceptives.	Clinical Trial	Total: 27 women Age: 19–47-years-old	Study 1: blood levels of angiotensin II were measured at intervals during the menstrual cycle in 3 women not treated with COC and during a single treatment cycle in 7 women who received 1mg of ethynediol diacetate and 0.1mg of mestranol (Ovulen) daily. Blood samples were collected after at least three hours of normal activity. Study 2: angiotensin II levels of 17 normal young women were measured before, during and after treatment with 1mg of EDA and 0.1mg of mestranol (Ovulen) or 2.5mg of Lynestrenol and 0.075mg of mestranol (Lyndiol). There were 10 women being studied for two months and 7 women for three months. In addition, the levels of PRS, PRA, and plasma renin concentration were measured in thirteen subjects before, during, and after COC therapy.	Angiotensin II: determined by extraction and radioimmunoassay. PRS, PRA, and renin concentration: kinetic enzyme bioassay procedures.	Study 1: In the 7 women treated with COC, blood levels of angiotensin II increased markedly and significantly ( *p* < 0.005) to about three times control levels. Ten days after stopping treatment, blood levels of angiotensin II had returned to the normal range. Study 2: In the 10 women treated with COC for two months, the levels of angiotensin II in the blood increased approximately three times above the baseline values (2.7 ± 1.0ng/100ml to 8.5 ± 2.9ng/100ml). After two months of treatment, mean values returned to normal in the third month and below baseline in the fourth month. In the 7 women treated with COC for three months, an identical pattern of response to angiotensin II was observed (mean increase to 8.6ng/100ml), with a fall to normal (2.6 ± 1.4ng/100ml) one month after discontinuation of treatment and values below baseline in the following month. Mean plasma levels of PRS, PRA, and angiotensin II in blood increased to 330%, 363%, and 314%, respectively, while the concentration of renin dropped to 52% of the control value. One month after the end of COC treatment, all values returned to normal.
De Leo et al. (2001) 14	To evaluate the clinical and hormonal effects of a new extra-low dose oral contraceptive, 15mcg EE and 60mcg gestodene, on RAAS in a group of 10 healthy women treated for 3 months, compared to a formulation containing the same hormones at a higher dose.	Clinical Trial	Total: 18 women Age: 35–39 years old	Group 1: 10 women used 15mcg EE / 60mcg gestodene (Arianna®, Schering). Group 2: 8 women used 20mcg EE / 75mcg gestodene (Fedra®, Schering). Blood samples were obtained before the study and after 3 months of contraceptive use for renin and aldosterone assay. In the first group, the sample was collected between days 18 and 21 of the menstrual cycle, between 8:30 and 9:30 am, after an hour of rest in the sitting position. In the second group, the sample was obtained during the third month of contraceptive use.	PRA and aldosterone: radioimmunoassay with Diasorin kits (DiaSorin SpA. Saluggia, Italy).	The contraceptive formulations studied showed insignificant changes in the value of PRA and aldosterone. Slight increase in PRA from baseline (16 ± 4% for group 2 and 18 ± 5% for group 1) and a slight reduction in plasma aldosterone levels (3.6 ± 0.5% and 4.6 ± 0.5%, respectively).
Kang et al. (2001) 15	Verify the effects of synthetic estrogens on RAAS activity and test the hypothesis that the use of COC decreases renin secretion.	Comparative observational cross-section	Total: 54 women Age: 25 ± 1 years	Study 1: 34 volunteers participated in the study, 15 COC users and 19 non-users. All were advised to adhere to a diet that maintained their normal caloric intake (sodium intake > 150mmol/day, protein intake 1–1.5g/kg/day) for 7 days prior to the study. Volunteers who were not COC users were studied in the follicular phase of menstruation. All COC users were studied between days 7 and 21 of their cycle, a period when they were ingesting 30mcg EE. Study 2: 20 volunteers participated in the study, 10 COC users and 10 non-COC users. Lower body negative pressure was used to activate RAAS by discharging arterial baroreceptors. Blood was collected at baseline for plasma norepinephrine, angiotensinogen, angiotensin II, plasma renin concentration, PRA, and aldosterone. Lower body negative pressure was incremental starting at 215 mmHg for 15 min, evolving to 225 mmHg for 15 min, and 240 mmHg for 15 min.	Angiotensin II: radioimmunoassay, in precooled tubes containing EDTA and angiotensinase inhibitor (0.1ml Bestatin solution, Buhlmann Laboratories). Aldosterone: radioimmunoassay, using the Coat-A-Count system. Angiotensinogen: measured indirectly by converting endogenous angiotensinogen to angiotensin I, then quantifying the amount of angiotensin I by radioimmunoassay. PRA: two-site immunoradiometry, where two monoclonal antibodies to human active renin are used.	Angiotensin II, PRA, angiotensinogen, and aldosterone increased significantly ( *p* < 0.05) at baseline in the oral contraceptive group. Incremental lower body negative pressure resulted in increases in most RAAS components, which were not significantly different between groups.
Giribela et al. (2015) 16	Evaluate the impact of a COC containing 20 mcg EE and 3 mg Drospirenone on BP, RAAS, insulin resistance, and androgenic profile of healthy young women using this COC.	Comparative observational	Total: 81 women Age: 18–40 years (average 30 ± 1)	49 healthy volunteers used COC containing 20mcg of EE and 3mg of drospirenone in a 24 day regimen of active pills followed by a 4 day break. 32 women with the same clinical characteristics who depend on male preservatives or non-hormonal intrauterine device were part of the control group.	PRA and aldosterone: enzymatic kinetic radioimmunological assay.	Significant increase in aldosterone (from 9.7 ± 1.0 to 20.8 ± 2.0; *p* = 0.0001) and in PRA (from 1.9 ± 0.2 to 4.2 ± 0.4; *p* = 0.0001).
Zakheim et al. (1976) 17	Studying the serum conversion of angiotensin I to enzymatic activity in women taking COC.	Comparative observational	Total: 22 women Age: 23.7 ± 1.1 for COC users; 26.5 ± 2.8 for non-COC users.	Enzyme conversion of angiotensin I, plasma angiotensin II, and BP were measured in 11 healthy women receiving oral estrogens ( norethindrone/mestranol, l7a-ethinyl-17-hydroxy-5(10)-estren-3-one and ethinylestradiol 3-methyl ether), 5 mg per day, for at least one year. The same measurements were performed in 11 healthy women who were not COC users and 11 healthy men of similar ages.	Enzymatic conversion of angiotensin I: modified Cushman and Cheung method. Plasma angiotensin II: radioimmunoassay kit provided by Schwartz-Mann Co (Orangeburg, N.J.) with the Boyd, Landon, and Peart procedure.	Plasma levels of angiotensin II increased significantly ( *p* < 0.05) in women on Enovid. Insignificant changes in the enzyme conversion activity of angiotensin I were observed.
Hollenberg et al. (1976) 18	To test the hypothesis that the use of COC is related to activation of the renin-angiotensin system, and that the responsiveness of the renal vasculature to angiotensin II provides an index of endogenous angiotensin II concentration in the vicinity of the renal vascular receptor.	Observational	Total: 86 women Age: < 49 years old	Data were available on renal blood flow and urinary sodium excretion for all volunteers. PRA measurements and angiotensin II levels were obtained for 19 subjects taking COC and 54 normal subjects not taking COC in identical clinical conditions.	Angiotensin II: measured by a dual antibody radioimmunoassay method. PRA: angiotensin radioimmunoassay.	PRS increased approximately 3-fold in association with a notable increase in circulating PRA ( *p* = 0.01) and angiotensin II levels ( *p* = 0.01) relative to sodium intake and excretion.
Cherney et al. (2007) 19	To examine the mechanisms by which women who are COC users maintain normal renal and systemic hemodynamic function, given the activation of RAAS.	Observational	Age: 18–49 years old	COC users were studied during the first 21 days of the menstrual cycle, and non-COC users were studied during the first 7 days of the menstrual cycle. Each study was performed after 7 days of a controlled diet consisting of 150mmol/day of sodium and 1.5 g/kg/day of protein. Compliance was determined by measuring 24 hour urinary sodium, potassium, and urea excretion on the 7th day. Blood samples were collected for inulin blank and for basal values of renin, PRA, angiotensin II, and aldosterone.	PRA: Two-site immunoradiometric assay, where two monoclonal antibodies to active human renin are used. Angiotensin II: radioimmunoassay. Aldosterone: radioimmunoassay, using the Coat-A-Count system. Angiotensinogen: measured indirectly by the endogenous conversion of angiotensinogen to angiotensin I.	Baseline aldosterone values ( *p* = 0.03) and angiotensin II levels ( *p* = 0.012) were high in women using COC. PRA levels were not significantly different between the two groups at baseline.
Oliveira et al. (2020) 7	To test the hypothesis that there is a difference between plasma renin levels in women who use and do not use COC, as well as determine its relationship to C-reactive protein.	Comparative observational cross-section	Total: 44 women Age: 20–30 years old	Group COC: 22 women using low-dose COC for at least one year. Low COC dose with 15-30mcg of EE associated with progestin was considered. Of the COCs used, 100% contained EE. 41% were associated with drospirenone, 27% with gestodene, 14% with levonorgestrel, 9% with chlormadinone acetate and 9% with desogestrel. Control Group: 22 women with the same characteristics without using COC for at least six months to one year. The collection of the control group was performed between the fifth and tenth day of the menstrual cycle, considering the smallest hormonal variations, and/or on the 28th day without medication (inactive phase).	Plasma renin: kinetic radioimmunoassay with EDTA plasma.	Women taking COC had higher serum renin levels (ng/ml/h) than women not taking this drug ( *p* < 0.01).

Abbreviations: BP, blood pressure; COC, combined oral contraceptive; EDA, Ethynodiol Diacetate; EE, ethinyl estradiol; LNG, Levonorgestrel; NET, Norethisterone (norethindrone); PAC, plasma aldosterone concentration; PRA, plasma renin activity; PRS, plasma renin substrate; RAAS, renin-angiotensin aldosterone system.

The risk of bias of clinical trials was assessed using the Cochrane Collaboration Tool, 10 as shown in Chart 2 . The quality of evidence from observational studies, assessed by the scale proposed by Downs and Black, 11 can be seen in its different domains in Chart 3 .

**Chart 2:** – Risk of bias by the Cochrane Collaboration Tool

Author, year	Random Sequence Generation	Allocation concealment	Blinding of participants and professionals	Blinding of outcome assessors	Incomplete outcomes	Selective outcome report	Other sources of bias
Straznicky et al. (1998) 6	High risk of bias	Uncertain risk of bias	Uncertain risk of bias	Uncertain risk of bias	Uncertain risk of bias	Uncertain risk of bias	Uncertain risk of bias
Briggs and Briggs (1982) 12	High risk of bias	Uncertain risk of bias	Uncertain risk of bias	Uncertain risk of bias	Uncertain risk of bias	Uncertain risk of bias risk	Uncertain risk of bias
Cain et al. (1971) 13	Uncertain risk of bias	Uncertain risk of bias	Uncertain risk of bias	Uncertain risk of bias	Uncertain risk of bias	Uncertain risk of bias risk	Uncertain risk of bias
De Leo et al. (2001) 14	Uncertain risk of bias	Uncertain risk of bias	Uncertain risk of bias	Uncertain risk of bias	Uncertain risk of bias	Uncertain risk of bias	Uncertain risk of bias

Source: Carvalho et al. (2013). 10

**Chart 3:** – Quality of evidence by the Downs and Black 11 scale

Author (year)	Communication External validity Internal validity: bias Confusion variable Power					Total
	(11 points)	(3 points)	(7 points)	(6 points)	(1 point)	(28 points)
Kang et al. (2001) 15	9	1	3	3	1	17
Giribela et al. (2015) 16	8	1	4	2	1	16
Zakheim et al. (1976) 17	7	1	4	2	1	15
Hollenberg et al. (1976) 18	8	2	4	3	1	18
Cherney et al. (2007) 19	9	1	5	2	1	18
Oliveira et al. (2020) 7	10	3	4	3	1	21

Source: Adapted from Downs and Black (1998). 11

## Discussion

This literature review concluded that the use of COC is associated with increased PRA, PRS, angiotensin II, and aldosterone in healthy women. This promotes increased activity of the RAAS, which may explain the prevalence of SAH in this population. 3 4

**Fig. 2:**
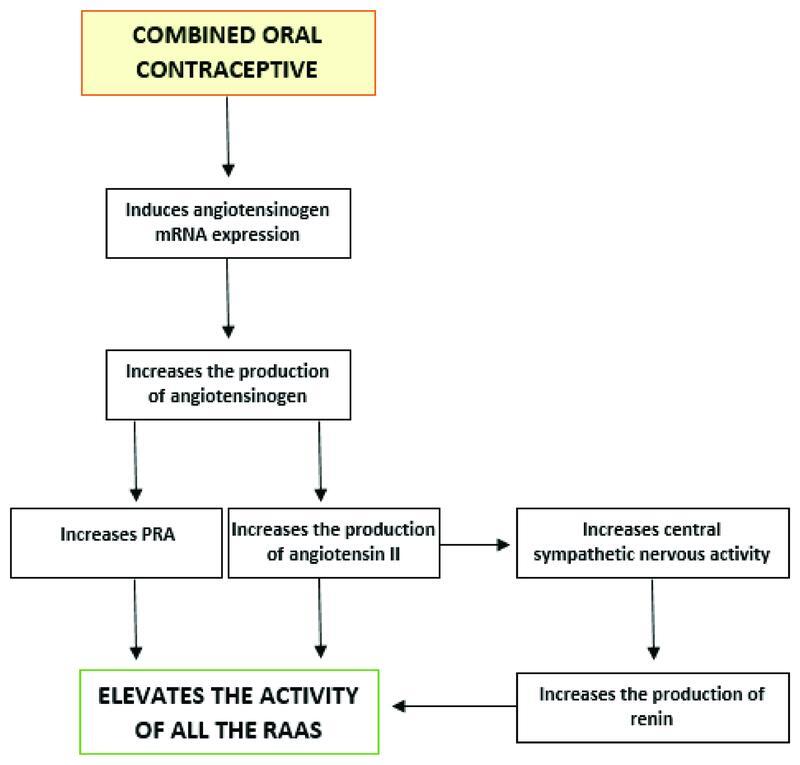
Mechanisms of action of the combined oral contraceptive on the renin-angiotensin-aldosterone system.

The RAAS is an important hormonal system, regulator of BP and electrolyte homeostasis of the organism. 20 The physiology of this system begins with the secretion of renin, an enzyme released by the renal juxtaglomerular apparatus that cleaves PRS, favoring the production of angiotensin I, later converted into angiotensin II by the action of the angiotensin converting enzyme (ACE). The results observed in studies point to an increase in PRS and PRA in women using COC. 6 7 12 13 15 16 18 One of the mechanisms that justify these findings is that ethinyl estradiol (EE), the synthetic estrogen that composes the COCs, is responsible for inducing the expression of angiotensinogen mRNA. 21 It also has the potential to increase the hepatic production of angiotensinogen, 21 which is accompanied by increased PRA. 4 Moreover, the elevated plasma renin and, consequently, angiotensin II values stimulate the central nervous system and increase sympathetic discharge. This increased sympathetic activity stimulates renal beta-adrenergic cells and favors a higher production of renin. 22 Thus, the mechanism of stimulation of the central nervous system feedbacks the production of renin and maintains the process of activation of the RAAS. Figure 2 presents the mechanisms by which the use of COC promotes greater activation of the RAAS.

In the same line of reasoning, Briggs and Briggs 12 developed studies aiming to test different dosages of COC in 137 women aged < 30 years. The PRA values and PRS concentration approximately doubled for the volunteers who were using the two contraceptives with higher EE dosage (50 mcg). In contrast, the renin concentration is reduced by about 30 to 40% of the pre-treatment values. Still in the same study, it was observed that lower doses of EE (30 mcg) resulted in smaller changes compared to higher dosages (50mcg), but the changes were still higher than the population not taking COC, with an increase of 20 to 30% in PRA, 12 to 20% of PRS, and a decrease of 15 to 20% in renin concentration. The decrease in renin concentration is justified by an increase in the levels of angiotensin II that decreases the production of renin through negative feedback, determining the balance of this system. 23 Despite this, the study developed by our research group found a median 2-fold higher renin concentration in women who use COC compared with women who do not. 7 The reasons that justify this opposition between the aforementioned studies are unknown. One hypothesis is the difference in the dosage and time of use of the COCs used in the studies, since the mean age of the selected samples were similar (23 years ^7^ vs 24 years ^12^ ). Possibly, the elevation of PRA is accompanied by a decrease in its concentration. However, the fact that our study had a lower EE dosage (15 to 30mcg ^7^ Vs 50mcg ^12^ ( with longer use (3 years ^7^ Vs 1 year ^12^ ), may have influenced the values of increased plasma renin oncentrations while their activity was normal. It is difficult to know because this has not been verified. This is a fertile field for future investigations.

Complementing the findings of the study by Briggs and Briggs, 12 evidence points out that PRS and PRA levels in women taking COC show elevations comparable to angiotensin II levels. 13 15 17 18 Cain et al. 13 evaluated 17 women taking COCs over a period of 2 to 3 months. Mean blood levels of PRS, PRA, and angiotensin II increased to 330%, 363%, and 314%, respectively, while renin concentration fell to 52% of the control value. 13 In this study, positive correlations were observed between PRA and angiotensin II (r=0.91), and between PRS and angiotensin II (r=0.73). 13 These correlations reinforce the idea that changes in the components of the RAAS during COC treatment are interdependent on the elevation of the PRS.

Angiotensin II is the molecule responsible for the majority of the physiological effects of the RAAS. It exerts its actions on target organs and can act by means of conversion into other molecules (angiotensin I–VII, angiotensin I–IX, or angiotensin III) or by binding to its AT1 and AT2 receptors. 23 24 In isolation, angiotensin II has the potential to stimulate the production of antidiuretic hormone, increase the reabsorption of sodium in the kidneys, stimulate sympathetic activity, and trigger direct vasoconstriction of the arterial vessels by binding to AT1 receptors, favoring the elevation of BP. 23 Additionally, when angiotensin II is converted to angiotensin III, it induces the production of aldosterone by the adrenals which will also act mainly on the kidneys. 23 Aldosterone is a mineralocorticoid, which by binding to the mineralocorticoid receptor on the epithelial cells, recruits sodium channels to the surface of renal epithelial cells, increasing sodium reabsorption, potassium excretion, and plasma volume expansion, culminating in BP elevation. 23 In a literature review carried out in 1996, COCs with high doses of EE (>30mcg) were identified as precursors of SAH, noting a high sodium retention effect in this population. 5

In this regard, so-called low-dose COCs (≤ 30 mcg of EE) have been formulated in an attempt to decrease the effects of EE on sodium retention. Additionally, the progestogenic components of COC have been recognized to antagonize the action of aldosterone (antimineralocorticoid effect) in the distal tubule of the kidney and, thus, increase sodium excretion. However, even in low-dose formulations and with the antimineralocorticoid effect, studies note an increase in aldosterone plasma levels, 14 15 16 19 being this is considered more as a compensatory mechanism than a hypertensive one. 16

It is important to elucidate that the outcomes evaluated may or may not impact clinical issues. We hypothesize that in the long term, the alterations caused in the RAAS lead to a greater development of cardiovascular diseases such as hypertension. The integrity of the arterial vascular endothelium is a key instrument in the regulation of RAAS. An intact endothelium favors the balanced production of RAAS substrates such as ACE. The balance of this system is essential for cardiac function, vasomotor tone, and vascular permeability, in addition to preserving blood fluidity. 25 26 In this aspect, the age of the volunteers is a relevant factor, since age is associated with the quality of the endothelial function. 25 However, only cohort studies or randomized clinical trials of a longitudinal nature will be able to confirm this hypothesis. So far, cohort studies have not found an association between COC use and risk of stroke, 27 myocardial infarction, 27 and all-cause mortality. 28

Finally, despite the observational studies in this review showing good methodological quality ( Chart 3 ), the clinical trials had uncertain methodological quality ( Chart 2 ). Future works that investigate this topic should seek to minimize the risk of bias so that the evidence is more robust, and the bases and biological mechanisms are better elucidated.

## Conclusion

Although new combinations with lower dosage and antimineralocorticoid action have been formulated to attenuate the adverse effects of COC, the results of this study suggest that COC promotes greater activation of the RAAS. Such conclusion supports the idea that its use provides, in the long and medium term, an increased risk of cardiovascular diseases and development of SAH.
